# A case report of monoarthritis in a COVID-19 patient and literature review: Simple actions for complex times

**DOI:** 10.1097/MD.0000000000026089

**Published:** 2021-06-11

**Authors:** Gilberto Cincinelli, Raffaele Di Taranto, Francesco Orsini, Andrea Rindone, Antonella Murgo, Roberto Caporali

**Affiliations:** a Department of Clinical Sciences and Community Health, Research Center for Adult and Pediatric Rheumatic Diseases, Università degli Studi di Milano; b Division of Clinical Rheumatology, ASST Gaetano Pini-CTO Institute, Milan, Italy.

**Keywords:** case report, COVID-19, inflammatory arthritis, psoriatic arthritis, reactive arthritis

## Abstract

**Rationale:**

COVID-19 presentation is multifaceted and up to 44% of patients affected by COVID-19 experience musculoskeletal complaints, mostly in the form of diffuse aspecific arthromyalgias. Nevertheless, only a few cases of arthritis following SARS-CoV2 infection are reported.

**Patient concerns:**

A 27-year-old man affected by nail psoriasis presented with monoarthritis 2 weeks after being diagnosed with COVID-19.

**Diagnoses:**

Diagnostic work-up and differential diagnosis were made difficult by patient isolation, absence of lab tests, and his visit via telemedicine, even though signs of first metacarpophalangeal joint involvement were clear.

**Interventions:**

Due to the inefficacy of acetaminophen and nonsteroidal anti-inflammatory drugs, the patient was prescribed oral steroids with a rapid benefit.

**Outcomes:**

The patient's response to oral steroid was prompt and maintained even after therapy tapering. Even so, a formal diagnosis was not possible due to a difficult diagnostic work-up and lack of a long-term follow-up.

**Lessons:**

Like many other viral diseases, SARS-CoV2 can play as a causative agent or as a trigger for inflammatory arthritis development in predisposed individuals.

## 1 Introduction

Since its outbreak, COVID-19 has reshaped the way health care services are provided.^[1]^ Its rapidity of spread among the population and potential evolution to a life-threatening disease are 2 key features of the burden SARS-CoV2 is exerting worldwide. Clinically, the main feature is pulmonary involvement leading to severe clinical features in almost 14% of the cases.^[2]^ Few such cases further worsen, with the development of a multi-organ disease fueled by a systemic inflammatory response called cytokine storm.^[3]^ However, the disease spectrum appears heterogeneous and like other cases of viral diseases, other organ systems can be clinically implicated.^[4]^ As discussed elsewhere,^[5]^ joint involvement ranging from arthralgia to chronic arthritis can accompany different viral infections. Although musculoskeletal complaints are relatively uncommon in endemic coronaviruses infections, up to 44% of patients affected by COVID-19 experience musculoskeletal complaints, mostly in the form of diffuse aspecific arthralgias and myalgias. In this paper, we discuss the case of a young man presenting with monoarthritis, compare it with similar cases published in the literature, and hypothesize on possible SARS-CoV2 roles in the development of inflammatory arthritis.

## 2 Case report

A 27-year-old man experienced acute swelling and pain of the first metacarpophalangeal (MCP) joint of the right hand 2 weeks after being diagnosed with COVID-19. He first managed his symptoms with Acetaminophen and oral nonsteroidal anti-inflammatory drugs (NSAIDs), ineffectively. His general practitioner (GP) prescribed him an oral steroid therapy, to be confirmed by a rheumatologist. The patient then contacted our rheumatology office and telephonically described his case. He stated that viral disease first presented with mild body temperature elevation (up to 37.3°C), flu-like symptoms, and mild, bilateral conjunctival injection, and was then submitted to a nasopharyngeal swab on the same day, which tested positive for SARS-CoV2. In absence of clinical features requiring hospitalization, the patient was thereafter isolated at home following local COVID-19 management guidelines.

**Figure 1:**
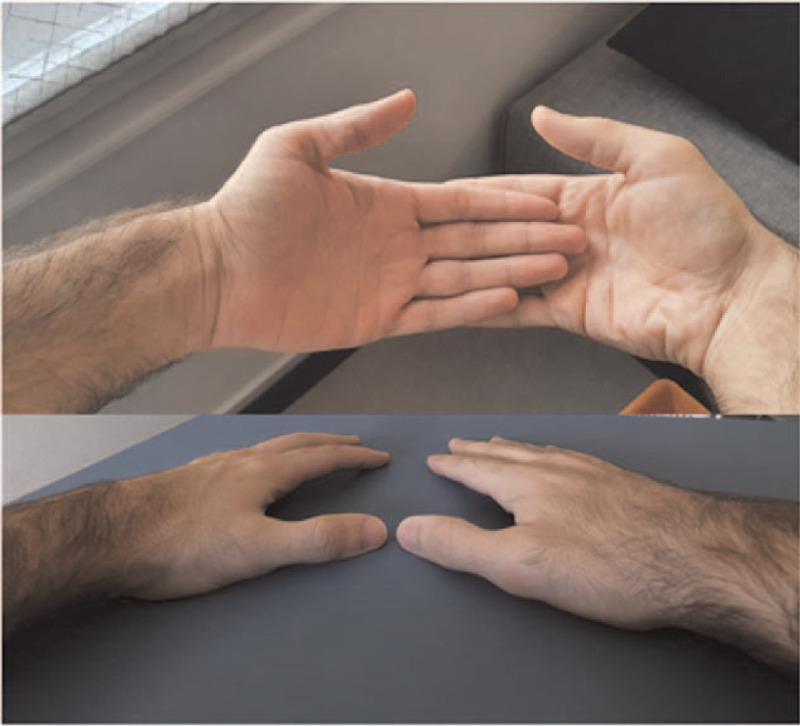
Swelling and redness of the first MCF of the right hand.

The patient reported that his viral illness symptoms had subsided 5 days before arthritis onset. He reported no previous trauma or articular overuse. On history, his overall clinical status was good, except for a diagnosis of nail psoriasis at a young age without overt psoriatic cutaneous or joint involvement. He denied recent genitourinary or gastrointestinal infections or new skin lesions. Unfortunately, neither recent blood tests nor imaging studies were available. Necessitating visualizing the case, we were able to get in touch with the patient via video call and evaluate his hands and wrists. On observation, redness and swelling of the first right MCP were easily distinguishable while no other sign of inflammation was remarked (Fig. 1).

**Figure 2:**
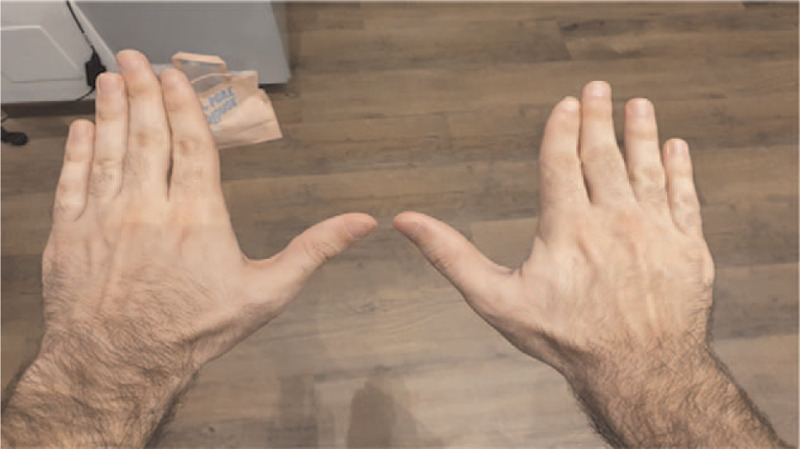
Reduction of swelling and redness of the first MCF joint of the right hand after 10 d of oral steroid therapy.

Due to the ineffectiveness of NSAIDs, we confirmed a short course of steroid therapy, starting with oral prednisone 10 mg/die with a rapid tapering schedule, confirming his GP decision. After steroid discontinuation, the patient reported the absence of pain or range of motion limitation and minimal residual swelling of the affected joint (Fig. 2). No other articular sites were subsequently affected by pain or swelling.

## 3 Discussion

This is the first case of arthritis affecting a COVID-19 patient not suffering from known rheumatological conditions we happened to handle in our Rheumatology unit. In the attempt to retrieve similar cases and their management, we searched PubMed using the terms “Covid-19”, “coronavirus”, “arthritis”, “reactive arthritis”. The search included all articles published in the English language until November 11, 2020. Only cases describing arthritis clinically defined by signs of joint inflammation (*dolor*, *rubor*, *calor*, *tumor*, *functio lesa*, either alone or in combination) or defined by imaging modalities (ultrasounds and/or magnetic resonance) were taken into account. 4 cases of arthritis arising in COVID-19 patients unaffected by prior rheumatological disease were retrieved and compared to our case.^[6–9]^

Our patient suffered from monoarthritis that occurred 15 days after being diagnosed with COVID-19. This temporal window is matched by the other published cases, where the time ranged from 8 days after probable exposition to 25 days after viral symptoms onset. Overall, arthritis presented as a mono- or oligoarthritis of the lower limbs, irrespective of COVID-19 severity, and showed a good and rapid response to therapy with systemic NSAIDs and/or intra-articular glucocorticoids. In contrast, arthritis in our patient developed in the upper limbs, and was unresponsive to oral NSAIDs, even though he experienced a dramatic and rapid response to a short course of oral steroid therapy at a moderate-low dose. Clinical and epidemiological characteristics of COVID-19 and COVID-19-associated arthritis are summarized in Table 1.

**Table 1:** Comparison between published cases of arthritis development following SARS-CoV2 infection and the case we present.

	Ono et al^[6]^	Parisi et al^[7]^	Saricaoglu et al^[8]^	Liew et al^[9]^	Present case
Age	50s	58	73	47	27
Sex	Male	Female	Male	Male	Male
Duration of COVID-19	21 d since swab positivity	25 d since prodromic symptoms	15 d since swab positivity	1 wk since exposition	15 d since swab positivity
Severity of COVID-19	Need for intubation	Need for hospitalization	Need for hospitalization	Nonsevere	Nonsevere
Number of joints involved	4	1	2	1	1
Localization	I MTP and I IP, bilaterally	Ankle	Ankles	Knee	I MCP joint
Imaging	No erosions or enthesophytes on plain X-rays	Synovial hypertrophy and power Doppler signal on ultrasound	Normal findings on radiographic examination	Suprapatellar effusion with mild osteoarthritic changes on X-rays	n/a
Synovial fluid analysis	Mild inflammatory fluid, negative cultures, and monosodium urate research	n/a	n/a	Turbid yellow fluid, no crystals, negative bacterial cultures and negative fluid PCR and culture for SARS-CoV2	n/a
Therapy	Oral NSAIDs and intra-articular corticosteroids	Oral NSAIDs	Oral NSAIDs	Oral NSAIDs and intra-articular corticosteroids	Oral steroids

IP = interphalangeal, MCP = metacarpophalangeal, MTP = metatarsophalangeal, NSAIDs = nonsteroidal anti-inflammatory drugs.

### 3.1 A case of reactive arthritis?

Reactive arthritis (ReA) is a sterile, often transient arthritis occurring after a distant mucosal infection and is part of the spondyloarthritis (SpA) spectrum. ReA pathogenesis is not fully characterized, but it seems that direct microorganism dissemination, molecular mimicry, and/or host response against infection operating in the context of a strong genetic background is key to disease development.^[10]^ The disease occurs 1 to 4 weeks after infection and affects more commonly young adults aged 20 to 40. Operating diagnostic criteria for ReA requires the presence of an asymmetric mono- or oligoarthritis, typically affecting lower limbs, associated with the evidence of a prior urogenital or gastrointestinal bacterial infection to label a diagnosis of “defined ReA”.^[11]^ However, criteria strictness regarding specific causative agents is not free from limitations. ReA concept is a recent object of revisiting, especially through the observed epidemiological reduction of sex-transmitted disease in high-income countries as well as recognition of miscellaneous pathogens responsible for the disease.^[12,13]^ Among miscellaneous responsible pathogens, some viruses are enlisted and the first COVID-19-related ReA cases start to emerge,^[10]^ as we aforementioned.

Due to our patient at-home isolation, we were unable to perform further investigations and a proper examination to thoroughly consider the differential diagnosis in inflammatory arthritis.

Ultimately, given its epidemiological concordance, clinical presentation, and the occurrence of a temporal-related event such as SARS-CoV2 infection, the possibility that might represent a case of reactive arthritis secondary to COVID-19 infection must be taken into strong consideration.

Complicating facts, our patient suffers from nail psoriasis, a condition predisposing to psoriatic arthritis (PsA) among patients with psoriasis.^[14]^ Back to the drawing board.

### 3.2 Viral triggers for PsA

Similar to most rheumatological conditions, inflammatory arthritis is the result of the combination of genetic susceptibility and environmental triggers.^[15–17]^ The latter encompasses various stimuli, among which viral infections are proposed as aetiological triggers for autoimmunity.^[18]^ This conclusion is drawn mainly from 2 observations. First, viral infections often precede the onset of autoimmune diseases.^[19]^ Second, different viral genomes have been retrieved from autoimmune diseases tissue targets, that is, synovial membrane in inflammatory arthritis, more frequently in rheumatoid arthritis and PsA.^[20,21]^

Postulated mechanisms for the role of viruses in autoimmunity genesis are diverse, such as molecular mimicry, bystander activation, and viral infection persistence.^[19,22]^

The hypothesis of infections as PsA trigger has come a long way, and many data were gathered in a recent systematic review.^[23]^ Most of our knowledge derives from data regarding Gram-positive and Gram-negative bacteria infections triggering PsA from psoriasis, most likely dysregulating the fragile immunological balance through Toll-like receptor (TLR)-2 and TLR-4 – mediated activation of the innate immune system.^[23,24]^ Not as strong as bacteria's, evidence for viral stimuli in the development of PsA mainly comes from the high prevalence of PsA in sub-Saharan patients with HIV infections.^[25]^ PsA susceptibility might be sustained by HIV pathogenesis, with the enrichment of IL-17+ CD8+ cells found in PsA synovium reflecting CD4+ T cells decrease and CD8+ T cells relative increase in HIV patients.^[26,27]^ Other viral diseases have been linked to PsA, with the strongest association being those with Parvovirus B19, cytomegalovirus (CMV), and Epstein-Barr virus (EBV), as they or their genome were found in a higher proportion of PsA synovium than ReA synovium.^[20,21]^ Once again, TLRs expressed by synoviocytes or other innate immune cells can be activated by antiviral compounds, notably TLR-3, -7, and -8, and may play a role in arthritis pathogenesis.^[28]^ Being affected by nail psoriasis, our patient is a subject susceptible to joint involvement. In the context of genetic susceptibility, SARS-CoV2 infection may represent the precipitating event to PsA onset.

### 3.3 Addressing COVID-19

**Figure 3:**
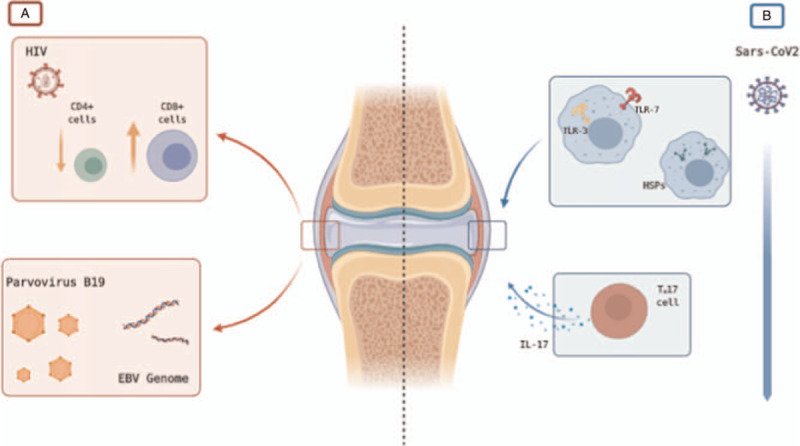
Comparison between (A) viral triggers in inflammatory arthritis and (B) possible mechanism behind COVID-19 induced inflammatory arthritis. (A) As discussed in the text, similarities between HIV-induced lymphocytes count alteration (i.e., decreased CD4+ cells count and a relative increase in CD8+ cells count) and inflammatory cells pattern in PsA synovium as well as the higher prevalence of HIV in sub-Saharan PsA patients fuel hypothesis on its role as a viral trigger for PsA.^[26]^ The presence of replicating Parvovirus B19 and EBV and CMV genome in RA and PsA synovium is another element supporting viral triggers in arthritis development.^[20,21]^ (B) Possible mechanisms through which SARS-CoV2 may serve as a trigger for inflammatory arthritis are dysregulation of TLRs activation,^[34]^ molecular mimicry with HSPs,^[41]^ and enhanced T_H_17 response^[31]^ as well as dysfunction of regulatory cells.^[35]^

Since its outbreak, different immune dysregulations have been found to correlate with COVID-19.^[29]^ Like HIV infection, lymphopenia is common among COVID-19 patients.^[30]^ Notwithstanding, in SARS-CoV2 related disease CD8+ cells subset is more susceptible to infection induced-depletion, as opposed to HIV and its immunopathogenesis in PsA development.^[31]^ TLR-3 and TLR-7 activation is presumed to be one of the first steps in SARS-Cov2 clearance.^[32,33]^ Nevertheless, the SARS-CoV2 infection relationship with TLRs is nothing but straightforward, as both clinical and experimental evidence seems to indicate that some unexplained TLRs alteration, either as inhibition or dysfunction, could sustain the defective immune response to viral infection and subsequent severe clinical manifestations.^[34]^ Despite the knowledge we possess about the viral role in inflammatory arthritis, COVID-19 appears to operate following different immunopathogenic pathways. Furthermore, other immunological alterations are observed in COVID-19 patients, such as regulatory cells dysfunction and increased circulating T_H_17 cytokines like IL-17 among all.^[31,35,36]^ Lack of regulatory function likely associated with an increase in circulating IL-17, whose axis is implicated in PsA pathogenesis,^[37,38]^ add complexity to the riddle of COVID-19 role in inflammatory arthritis development. Last but not least, human-viral molecular mimicry is supposed to be responsible for the multifaceted phenotype of COVID-19 disease.^[39]^ Different autoimmune neurological and hematological conditions encountered as COVID-19 complications have been spotted as potential molecular mimicry results.^[40]^ Among these, some cases of Guillain-Barré syndrome are supposed to bear a viral similarity to heat shock proteins (HSPs) as pathogenic mechanism.^[41]^ HSPs are ubiquitous intracellular proteins that serve as chaperon proteins, MHC-peptide binding, and protein synthesis and folding.^[42]^ They are upregulated in inflammatory states and are thought to balance immune tolerance.^[43]^ However, these proteins are immunogenic and when inflammatory stimuli overcome regulatory factors, HSPs may aid break the tolerance.^[43]^ In inflammatory arthritis, HSP-60 and HSP-70 are up-regulated and appear to stimulate synovial and peripheral T cell proliferation and activation.^[44]^ Lucchese and Flöel^[41]^ demonstrated that SARS-CoV2 shares an immunologically relevant sequence of peptides with HSP60. Though the same sequence of peptides is implicated in loss of immune tolerance in demyelinating diseases and not in inflammatory arthritis,^[45]^ this association reduces the theoretical gap between COVID-19 and the development of inflammatory arthritis (Fig. 3).

## 4 Conclusion

COVID-19 pandemic is posing difficulties in the management of patients both in hospital settings and territorial offices. Whether affected by chronic diseases or presenting with acute conditions not requiring urgent evaluation, many patients are assessed via telemedicine, that is, the remote delivery of healthcare services. This approach can help provide valuable solutions to the patients’ problems. One of the prices is the risk of leaving nonurgent complex cases and further investigations temporarily unresolved or postponed. Such is the case for our patient, whose resolutions of joint symptoms may serve as a temporal bridge for when an appropriate diagnostical work-up will be feasible to label this case either as reactive arthritis or psoriatic arthritis. Simple actions for complex times.

## Author contributions

**Conceptualization:** Gilberto Cincinelli, Roberto Caporali.

**Supervision:** Roberto Caporali.

**Writing – original draft:** Gilberto Cincinelli, Raffaele Di Taranto, Francesco Orsini, Andrea Rindone.

**Writing – review & editing:** Gilberto Cincinelli, Antonella Murgo, Roberto Caporali.
